# Factors Influencing Primary Healthcare System in the Achievement of Universal Health Coverage in the WHO‐AFRO Region: An Integrative Review

**DOI:** 10.1002/puh2.70081

**Published:** 2025-07-18

**Authors:** Emmanuel O. Adesuyi, Opeoluwa O. Olabode, Oluwatosin C. Olarinde, Blessed O. Oyama, Inioluwa O. Aderemi, Gloria O. Alao, Bose C. Ogunlowo

**Affiliations:** ^1^ College of Health and Care Professions Birmingham City University Birmingham West Midlands UK; ^2^ Institute of Nursing Research Osogbo Nigeria; ^3^ University of Ilesa Ilesa Osun Nigeria; ^4^ Afe Babalola University Ado Ekiti Nigeria; ^5^ South Tyneside and Sunderland, NHSFT South Shields UK; ^6^ Department of Community Health Nursing Obafemi Awolowo University Ile‐Ife Nigeria

**Keywords:** primary health centre, universal health coverage, WHO‐Afro

## Abstract

**Background:**

The primary health centres are designed to be the first line of contact with the public and the main target for achieving universal health coverage (UHC). However, despite global advancements in healthcare, Africa continues to face unacceptably high health outcome indices, underscoring the significant challenges in achieving UHC. This review synthesises evidence on the factors influencing the primary healthcare system, focusing on its role in achieving UHC within the WHO‐Afro region.

**Methods:**

This is an integrative literature review. An extensive and systematic search for eligible literature was performed in eight databases between 2000 and 2024, including APA PsycINFO, CINAHL, Medline, Scopus, EmCare, Embase, Ovid Nursing and Gale Academic One File. The articles identified were critically appraised. A narrative synthesis of evidence was done to fully explore the review question. The findings were presented using Preferred Reporting Items for Systematic Reviews and Meta‐Analyses (PRISMA) in‐line with EQUATOR guidelines.

**Result:**

Overall, 26 studies were included in the review. Six themes captured the factors influencing the PHC system in the achievement of UHC: (1) physical access to critical services, (2) availability of resources, (3) sociodemographic and cultural factors, (4) access to data and health information, (5) legislation and policy and (6) quality of care.

**Conclusion:**

This review highlights interconnected factors necessitating a holistic approach to optimising PHC effectiveness and driving progress towards achieving UHC. Integrating human, financial and infrastructural resources alongside community‐driven strategies, equitable digital technologies and robust policy frameworks is essential to improving health outcomes and achieving UHC.

## Graphical abstract

Of the 26 studies included in the review, six interconnected themes captured the factors influencing the PHC system in the achievement of UHC: physical access to critical services, availability of resources, sociodemographic and cultural factors, access to data and health information, legislation and policy and quality of care.

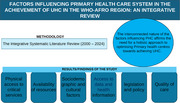

## 1 Introduction

Despite the advancement of healthcare services globally, low‐ and middle‐income countries (LMICs) health outcome indices remain unacceptably high; there have been surges recorded in the mortality and morbidity rates of specific diseases, and these are particularly dominant in the World Health Organisation (WHO) African regions [1]. In furtherance, based on the latest data generated by the United Nations Population Division, the life expectancy age at birth of an average Nigerian for both sexes is the lowest (54.64) on a global level, as compared to other countries of the world [2]. According to WHO [3], most of the world's population still spends out‐of‐pocket for healthcare, especially in Sub‐Saharan Africa (SSA), due to significant disparities in service accessibility. An effective and efficient primary healthcare system could have prevented this situation. These statistics indicate the state of the population's health, and this situation can only be improved with equitable access to the healthcare system through universal health coverage (UHC).

The primary health centres are designed to be the initial and the primary point of contact with the general public and the main target for achieving health for all [4, 5]. UHC is one of the tools for achieving sustainable development goals by 2030, with notable records of countries that have begun to assimilate the SDGs, with the state authorities making efforts to localise actions, though at varied rates [6]. Hence, evidence suggests that expanding primary healthcare (PHC) programmes in LMICs could save more lives and raise average life expectancy [4]. Similarly, based on evidence from pieces of literature, PHC is viewed as a cornerstone for strengthening health systems [7, 8, 9].

Despite the essentiality of this system, many WHO‐Afro countries are still lagging in having an efficient and effective primary healthcare system; people no longer use the primary health centres in this region due to a myriad of reasons, including inequitable distribution of resources, lack of skilled personnel and inadequate facilities for proper operation among others. Even though patient contact with the secondary and tertiary parts of the healthcare system may have depended on a robust referral system from the primary health centres, this explains why the achievement of UHC and sustainable development goals has become a herculean task [10, 11, 12, 13]. In light of these challenges, and because of the need to strengthen the primary healthcare system in achieving UHC, we conducted this review to summarise evidence relating to the factors influencing the primary healthcare system in the achievement of UHC across the WHO‐African region.

## 2 Methodology

This integrative systematic literature review was conducted on the basis of the seven‐step framework suggested by authors [14, 15, 16, 17]. They include (1) writing the review question, (2) determining the search strategy, (3) critical appraisal of the search results, (4) summarising the search results, (5) data extraction and reduction, (6) analysis and (7) conclusions and implications. Researchers have argued that integrative reviews should be held to the same quality standards as primary research [16, 17, 18]. This might be because it is systematic and integrates literature from diverse sources and methodologies to address specific research questions [19, 20]. The review protocol was registered with the Open Science Framework (OSF) network (https://doi.org/10.17605/OSF.IO/XW6TS).

### 2.1 Writing the Review Question

The primary review question is as follows: What are the factors influencing the PHC system in the achievement of UHC across the WHO‐Afro Region?

### 2.2 Determining the Search Strategy

A general search was performed on Google Scholar to scope the field and articulate plans for specific database searches. The result revealed that the phenomenon of interest was investigated in several studies using various methodologies. The search strategy was initiated by refining the research questions and applying the SPIDER framework (sample, phenomenon of interest, design, evaluation and research type) proposed by Cooke et al. [14] to structure and detail the search terms, as shown in Table 1.

**TABLE 1:** Review question model using SPIDER framework [14].

S (Sample)	Primary health centres in Africa
PI (Phenomenon of interest)	Determinants and factors influencing
D (Integrative)	Integrative
E (Evaluation)	Universal Health Coverage, effectiveness efficiency
R (Research type)	(Quantitative, qualitative and mixed method)

We established a team of four researchers with diverse backgrounds and expertise to conduct the literature search. Keywords articulated from the initial search were used to search relevant databases. The databases used to identify articles relevant to this study include EBSCO, which was used to search APA PsycARTICLES, CINAHL Ultimate and Medline. ProQuest was used to search ProQuest Dissertations. Ovid was used to search EmCare, Embase and Ovid Nursing Database. We also searched Scopus and Gale Academic One File to ensure crucial publications were not omitted. According to Whittemore and Knafl [3], conducting a systematic and comprehensive search is essential for ensuring the rigour and credibility of the review process.

#### 2.2.1 Search Terms

The following keywords were modified to match the specific database using appropriate Boolean operators. Determinants*, factors*, ‘factors influencing’, Primary Health Centres, PHCs*, African countries, WHO‐Afro region*, universal health coverage, UHCs*, UHC*. (Determinants of an effective and efficient primary health care system)* OR (Factors influencing PHCs)* AND (Low and middle‐income countries)*. (Determinants of an effective and efficient primary health care system)* OR (Factors influencing PHCs)* AND (Africa)* AND universal health coverage. ‘Primary health care AND Africa AND Universal Health Coverage’. The classification of countries into the WHO‐Afro region is based on the guidance provided by WHO [21] (https://www.afro.who.int/countries).

#### 2.2.2 Screening and Eligibility Criteria

We used Mendeley Library to manage and remove duplicate articles identified through database searches. Articles were initially screened by the abstract and topic of articles using the eligibility criteria (see Table 2). Articles were then screened for full‐text accessibility and conformity to the eligibility criteria. The final selection was made after critically appraising the articles for quality and methodological strength. Findings from the critical appraisal were reported in the results and discussed in the discussion part of this assignment. The inclusion and exclusion criteria that formed the eligibility criteria used for selecting articles for this study are shown in Table 2.

**TABLE 2:** Inclusion and exclusion table.

Criteria	Inclusion	Exclusion
Phenomena	Determinants, factors influencing primary health centres in Africa	Any article that is not focused on the included phenomena of interest
Geographical location	All 47 WHO‐Afro countries included in (https://www.afro.who.int/countries)	Any study carried out on participants outside Nigeria
Year of publication	Articles published between 2000 and 2024	Articles published before 2000
Language	All articles published in English and French	English and French are the official language in WHO‐Afro countries, and it is unlikely to have articles published in other language

### 2.3 Critical Appraisal of the Search Results

Articles selected for this review were examined for quality using the Johanna Briggs Institute, JBI, checklists for quantitative and qualitative evidence [22, 23]. Critical appraisal, also known as the risk of bias assessment of included studies, is a crucial step in conducting a reliable systematic review [22]. The checklist for appraising quantitative evidence consists of 8 questions, whereas the one for appraising qualitative evidence consists of 10 questions requiring the answers yes, no, unclear or not applicable in both cases [24]. The appraisal results for the articles selected for this review are presented in Tables  3 and 5 .

**TABLE 3:** Critical appraisal of selected quantitative studies.

Authors	Q1	Q2	Q3	Q4	Q5	Q6	Q7	Q8
Ramadan et al. [25]	Y	Y	U	Y	Y	Y	Y	Y
Macarayan et al. [26]	Y	Y	Y	Y	Y	Y	Y	Y
Global Monitoring Report [27]	U	U	U	U	Y	Y	Y	U
Mutono et al. [28]	N	Y	Y	Y	Y	Y	Y	Y
Simen‐Kapeu et al. [29]	N	U	Y	U	Y	Y	Y	U
Makinde et al. [30]	N	U	Y	Y	Y	Y	U	U
Alhassan et al. [31]	Y	NA	U	Y	Y	Y	Y	Y
Adewole et al. [32]	Y	Y	U	Y	Y	N	Y	U
Karamagi et al. [33]	N	U	Y	Y	Y	N	Y	Y
Bresick et al. [34]	Y	Y	Y	Y	Y	Y	Y	Y
Arhin et al. [35]	N	U	Y	U	Y	Y	Y	Y
Gebremichael et al. [36]	Y	Y	U	Y	Y	Y	Y	Y
Ekenna et al. [37]	N	Y	Y	Y	Y	Y	Y	Y
Bolongaita et al. [38]	U	U	U	Y	Y	Y	Y	Y
Vallières et al. [39]	U	Y	Y	Y	Y	Y	Y	Y

*Note:* This table shows the result of the critical appraisal of quantitative studies selected article using JBI (2020) guideline. Q1 = Were the criteria for inclusion in the sample clearly defined? Q2 = Were the study subjects and the setting described in detail? Q3 = Was the exposure measured in a valid and reliable way? Q4 = Were objective, standard criteria used for measurement of the condition? Q5 = Were confounding factors identified? Q6 = Were strategies to deal with confounding factors stated? Q7 = Were the outcomes measured in a valid and reliable way? Q8 = Was appropriate statistical analysis used?. Y = yes, N = no, U = unclear and N/A = not applicable.

### 2.4 Summarise the Search Results

**FIGURE 1:**
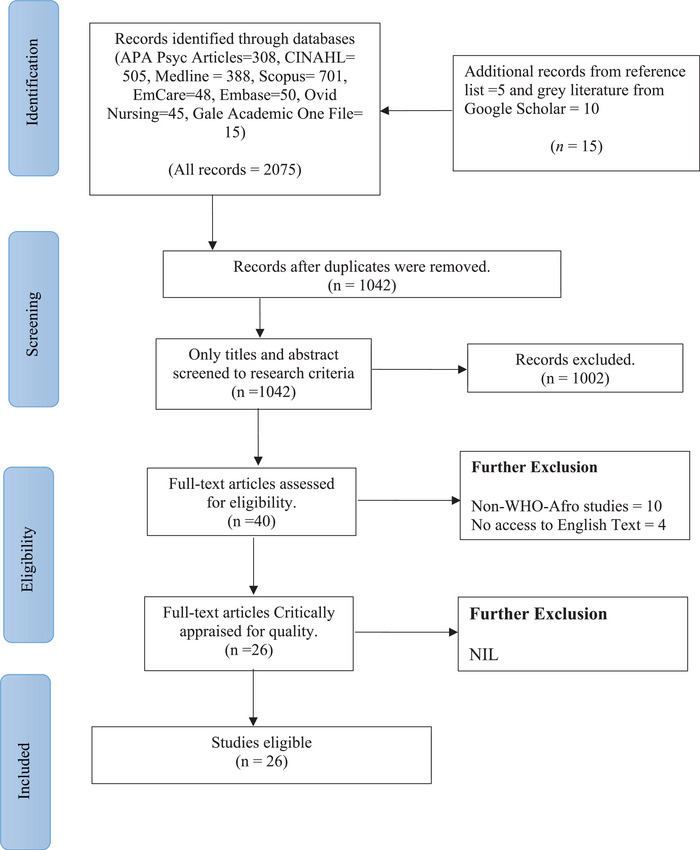
PRISMA flow chart of review result (2000–2024).

The initial search yielded 2075 articles across eight databases and two additional sources between 2000 and 2024. Mendeley was used to manage the references and remove duplicates. After removing 1033 duplicates, the remaining articles were made to go through screenings using title, abstract and full text, including the eligibility criteria. At the end of the screening, 26 articles were selected for the review. The Preferred Reporting Items for Systematic Reviews and Meta‐Analyses (PRISMA) Flow Chart, which is the JBI standard for reporting systematic reviews, was used to present the search results, as shown in Figure 1.

### 2.5 Data Extraction and Reduction

Key data relevant to the focus of this review were extracted from the selected studies using JBI templates for data extraction in integrative systematic reviews [40]. An overview of the whole article was extracted, including the author's name, publication year, article title, study aims, study design or method, data collection method, strength/weakness and key findings. This is shown in the data extraction table (see Table 4). The data required to answer the review questions were then extracted from each article's method, results, discussion and conclusion in a synthesis‐compliant format.

**TABLE 4:** Data extraction table.

Reference/Title	Study aim	Study design	Data collection method	Strength and weakness of study	Findings relevant to the review
Adewole et al. [32]/Geospatial distribution and bypassing health facilities among National Health Insurance Scheme enrollees: implications for universal health coverage in Nigeria	To assess the geospatial distribution and access to healthcare facilities under the National Health Insurance Scheme (NHIS) of Nigeria	Quantitative descriptive cross‐sectional study	Interviewer‐administered questionnaire to 420 participants	The current study offers insights that would be beneficial for evidence‐based decision‐making policies in health planning and effective allocation of health resources based on population needs. The current scope could not accommodate the cause for bypassing reported in this investigation. The real participant's residence addresses were 300–500 m apart from the landmarks utilized as proxies. The use of landmarks as proxy for participants’ home locations is acknowledged to have an impact on distance calculations	PHC facilities were distributed more equitably than secondary and tertiary institutions across all LGAs in the research region. PHC institutions were not contracted by the NHIS to offer services to registrants, yet they had the most equal geographic dispersion of the three levels of care. Despite the provision of broad PHC facilities in both urban and rural regions, these centres were not used as service providers under the NHIS
Assan et al. [41]/Challenges to achieving universal health coverage through community‐based health planning and services delivery approach: a qualitative study	To explore the challenges to achieving universal health coverage (UHC) through the community‐based health planning and service (CHPS) initiative in Ghana	Qualitative study design	Face‐to‐face in‐depth interviews of 67 participants	The stratified data collection strategy and the variety of study participants from across different levels and settings helped gain a broader understanding of the phenomenon. By investigating several components of the community‐based health planning and service effort, the findings contribute to understanding of the phenomena from a variety of disciplines	On the basis of our findings, inadequate understanding of the community‐based health planning and service (CHPS) initiative concept, major contextual changes with stalled policy change to meet growing health demands, and changes in political landscape and leadership with changed priorities threaten CHPS sustainability
Gebremichael et al. [36]/Impact of good governance, economic growth and universal health coverage on COVID‐19 infection and case fatality rates in Africa	To assess the impact of good governance, economic growth and UHC on the COVID‐19 infection rate and case fatality rate (CFR) among African countries	Analytical ecological study design; a cross‐sectional study	Data were extracted from publicly available databases (i.e., Worldometer, Worldwide Governance Indicators, Our World in Data and World Health Organisation (WHO) Global Health Observatory Repository)	Wide scope as all 54 African countries were covered by this study Because of the ecological study design, it is difficult to make a clear association between dependent and explanatory variables to draw conclusions, as the data are used in aggregate rather than for individual patients	Strengthening governance standards and ensuring economic growth are required to lessen the impact of pandemics on people in African countries to achieve UHC
Karamagi et al. [33] [Cross county lessons sharing on practices, challenges and innovation in primary health care revitalisation and universal health coverage implementation among 18 countriea in the WHO African Region.	To present an approach for understanding the functionality of health systems that provide actionable information to decision‐makers	Quantitative	Data from 18 WHO‐Afro regions were collected from data sources that provide primary data, including household surveys and facility assessments; publicly available sources, including the WHO Health Observatory, the United Nations Sustainable Development Goals database and the World Bank World Development Indicators	It uses a systematic, easily replicable method based on publicly available data to provide country‐specific information on where to focus on system development. Areas of the health system where interventions are needed were not mentioned, and the index with exogenous policy changes over time was not correlated to evaluate its influence on policy change at country level	Access to vital services has the lowest capacity in most nations in the area, owing to limited physical access to services. In comparison to other sources, public and out‐of‐pocket funding amounts are the best indicators of system functionality
Global Monitoring Report [27]/Tracking Universal Health Coverage	To analyse progress towards and impediments to achieving UHC	Quantitative	A formal country consultation was conducted between mid‐March and the end of June 2021 with nominated focal points from national governments and national statistical offices. Data were also collected from existing WHO and United Nations agency modelled estimates, country reported data, and published results from household surveys	The result provides an extensive analysis of nation‐wide indices. However, it might not be able to capture minute but crucial issues at the individual level	The COVID‐19 pandemic has resulted in major interruptions to the supply of crucial health services. Rising poverty and falling earnings as a result of the global economic downturn are projected to create financial obstacles to care access and financial hardship due to out‐of‐pocket health expenses for people seeking treatment, particularly among disadvantaged groups. The pre‐COVID obstacles, together with the added difficulties posed by the epidemic, make the search for UHC even more urgent
Ray and Mash [42]/Innovation in primary health care responses to COVID‐19 in Sub‐Saharan Africa	To synthesise the lessons and ideas relating to PHCs to address the challenges raised by the COVID‐19 pandemic	Qualitative descriptive design	A thematic document analysis of short reports published in African Journal of Primary Health Care and Family Medicine (PHCFM) with a thematic analysis. All short reports accepted for publication in a special collection on COVID‐19 were included in the qualitative analysis	The study was descriptive and did not present empirical data. They did not report on research or provide definitive evidence of effectiveness of the ideas presented	Eight major themes were derived from the data: community‐based activities; screening and testing; reorganisation of health services; emergency care for COVID‐19; maintenance of essential non‐COVID‐19 health services; caring for the vulnerable; use of information technology and reframing training opportunities
Tumusiime et al. [43]/Building health system resilience in the context of primary health care revitalization for attainment of UHC: proceedings from the Fifth Health Sector Directors’ Policy and Planning Meeting for the WHO African Region	The 2‐day forum focused on building health system resilience to facilitate service continuity during health threats, PHC revitalisation and health systems strengthening towards UHC	Qualitative research method	Data were collected through group discussion based on specific predetermined questions and then an open‐ended conversation in plenary	Inability to record objective observations is a weakness	Four main issues were raised: (1) multisector/intersectoral collaboration, (2) transitioning from fragmentation to integration, (3) assuring implementation and information exchange and (4) rethinking resilience and embracing antifragility
Kredo et al. [44]/Primary care clinical practice guidelines in South Africa: qualitative study exploring perspectives of national stakeholders	To explore the perspectives of national stakeholders on primary care clinical practice guidelines in South Africa	Qualitative method	Data were collected through semi‐structured, in‐depth interviews with 37 participants	The inclusion of members from different disciplines who could point to gaps in the process was a strength. One weakness is response bias as those interviewed were all active members in CPG development and likely to be positively inclined towards the value of the work	Medical schemes, professional societies, at times with pharmaceutical industry support, NGOs and provincial hospital or clinic level initiatives all develop CPGs where no or limited guidance exists to address gaps in clinical guidance not covered by NDoH However, PHC providers such as nurses play the central role in service delivery at PHC they are not seen to be driving CPG activities nationally and were described as noticeably absent in leading roles, except perhaps in consultation processes or external reviews of documents
Michel et al. [45]/What we need is health system transformation and not health system strengthening for universal health coverage to work: Perspectives from a National Health Insurance pilot site in South Africa	To present the perspectives of respondents in response to the research question ‘what do you think needs to happen for the current UHC policies to be implemented successfully, and why?’	Qualitative	In‐depth face‐to‐face interviews of key informants (*n* = 71)	Triangulation and comparison of policy views across the different levels of the health system provided a balanced view of what coal face actors think needs to be done for UHC to work. One weakness is that the study took place in one pilot district	Themes that emerge includes ‘Make primary healthcare work’, ‘Context‐sensitivity and flexibility needed from supervisors’, ‘Transform the way that policies are implemented’, ‘Involve change agents’, ‘Public–private partnerships: Learning from the private sector’, ‘Transform processes and systems’, ‘Plan for equipment servicing and have clear maintenance plans to save costs and improve outcomes’, ‘Leadership is key’, ‘Take epidemiological transitions into account’, ‘Adopt a systems lens’, ‘Streamline data collection for efficiency and maximising data use in planning and evaluation’
Bresick et al. [34]/Evaluating the performance of South African primary care: a cross‐sectional descriptive survey	To evaluate the quality of South African primary care using the Primary Care Assessment Tool (PCAT)	Quantitative descriptive cross‐sectional survey	Data were collected via survey from 413 patients, 136 health workers and 55 managers were analysed from 30 community health centres across four provinces of South Africa	The survey also excluded clinics, and sampling was based on the needs of an observational study designed to compare facilities with and without family physicians. It is difficult, therefore, to generalise the findings to primary care as a whole in South Africa	Patients rated comprehensiveness, coordination, cultural competency and the availability of the PHC team as reasonably strong aspects of primary care (≥66% or more scoring as ‘acceptable or good’). Managers and providers agreed with them but were also much more positive about the comprehensiveness of services and availability of PHC team members Patients remain dissatisfied with accessibility, and only 50% use their primary care service as first contact
Simen‐Kapeu et al. [29] /Galvanizing Action on Primary Health Care: Analyzing Bottlenecks and Strategies to Strengthen Community Health Systems in West and Central Africa	To identify common community health system bottlenecks, review progress made by selected countries, and propose strategies to move forward	Quantitative	Systematic analysis of bottlenecks to strengthen community health systems and quantitative analysis of country profiles with selected tracer indicators	Although this first regional assessment provides further insights into challenges and strategies to strengthen community health systems, further research linking the reduction of bottlenecks and outcomes is warranted	14 countries reported poor MNCH/HIV/TB service integration as a severe bottleneck. Poor community engagement was also identified as a severe bottleneck in most countries. Most country teams self‐reported that the supply chain was a severe health system bottleneck due to reasons such as lack of funding, including for operating costs; ineffective procurement cycles and delays; inadequate commodity security strategies and inadequate quantification for CHWs
Makinde et al. [30]/ Distribution of health facilities in Nigeria: Implications and options for Universal Health Coverage	To assess the geographic and sectoral distribution of health facilities in Nigeria. To discuss how the distribution of health facilities may influence or affect the UHC strategy adopted	Quantitative	The Nigerian Master Health Facility List (MFL) was used to gather information on the names of all the registered health facilities in the country, state of location, local government area, ownership status, the level of care and their unique national identifier	The emphasis of the study has been on the physical availability of the health facilities but has not taken into account the functionality of these facilities because of limited data in the MFL. There may have been facilities that have closed out and others that have been registered with authorities since this list was completed in 2013, thereby making this list inaccurate	Primary health facilities are the most physically accessible level of healthcare, providing basic healthcare services to the largest proportion of the population and accounting for 30,345/34,423 (88%) of health facilities in the country. In some southern states, there were at least 20 private health facilities per 100,000 of the population. This is in sharp contrast with states in the Northwest and North East of the country where private health facility availability was below 0.3 per 100,000 of the population for some states
Mutono et al. [28]/Impact of traffic congestion on spatial access to healthcare services in Nairobi	To assess the impact of traffic congestion on access to healthcare facilities and to the healthcare professionals across the healthcare facilities	Quantitative	Data were collected from the Federal Ministry of Health database in Kenya. A total of 944 primary healthcare, 94 secondary healthcare and 4 tertiary healthcare facilities were mapped out in Nairobi County. Traffic probe data were also used to identify areas within a 15‐ to 45‐min drive from each of the health facility during the peak and off‐peak hours	A weakness of the 2SFCA method was the unavailability of a standard metric on the minimum time that one ought to travel while seeking healthcare services and the ratio of healthcare professionals to population, making it difficult to compare study findings from different countries or populations	The proportion of the population within the catchment areas of each level of healthcare that could access the healthcare facilities within ≤15, 30 and 45 min during off‐peak hours was significantly reduced during peak traffic times. Only secondary health facilities were accessible to the whole population during peak and off‐peak hours at 45 min threshold. Traffic congestion played a significant role on the proportion of population able to access the different levels of healthcare with more than a third of the population not able to access health facilities within 30 min drive time. There was also sub‐optimal access to healthcare professionals
Ameh et al. [46]/A qualitative inquiry of access to and quality of primary healthcare in seven communities in East and West Africa (SevenCEWA): perspectives of stakeholders, healthcare providers and users	To describe the viewpoints of healthcare users, healthcare providers and other stakeholders on health‐seeking behaviour, access to and quality of healthcare in seven communities in East and West Africa	A qualitative study was conducted in 4 Nigerian communities and one community each in Kenya, Uganda and Tanzania	A purposive sampling technique was used to recruit 155 respondents to 24 focus group discussions, 25 healthcare users, healthcare providers and stakeholders for in‐depth interviews and 11 healthcare providers and stakeholders for key informant interviews	Only one co‐author coded the data, and this could lead to bias or misinterpretation of data	Access to primary healthcare in the seven communities was limited, especially use of health insurance. Quality of care was perceived to be unacceptable in public facilities, whereas cost of care was unaffordable in private facilities. Health providers and users as well as stakeholders, highlighted shortage of equipment, frequent drug stock‐outs and long waiting times as major issues but had varying opinions on satisfaction with care
Kumar et al. [47]/Mapping services at two Nairobi County primary health facilities: identifying challenges and opportunities in integrated mental health care as a Universal Health Coverage (UHC) priority	To understand facility‐level barriers and facilitators to the integration of mental health into routine care for adolescents in health centres in Nairobi County, Kenya	A qualitative study	Twelve (12) healthcare workers (HCWs) were enrolled for this study. The two facility in‐charges were selected due to their administrative rank, whereas the other 10 HCWs were invited to participate from different clinics	Although the study offers valuable insight, it is impossible to generalise the findings as participants were purposively selected during their free period for the qualitative study	The study identified burnout in health professionals due to understaffed facilities with overly increasing patient turn‐ups. Healthcare facilities provided several free health services, daily outpatient services, weekly mental health services and weekly clinics. However, there was poor knowledge about mental health policy or legislature as well as standard facility‐based or public information protocols for mental healthcare
O'Brien et al. [48]/Strengths, Weaknesses, Opportunities, and Threats Analysis of the Use of Digital Health Technologies in Primary Health Care in the Sub‐Saharan African Region	To expand on previous research on the subject and to specifically evaluate the current strengths and weaknesses, as well as the main opportunities and threats to implementing DHTs in primary healthcare in Sub‐Saharan Africa (SSA)	A combination of qualitative approaches was used (i.e., web‐based focus groups and semi‐structured interviews)	17 members of the African Forum for Primary Care (AfroPHC) were invited to participate in the focus groups. 40 out of the 53 countries in Africa	Although the study offered valuable insights from various professionals, there was a potential lack of transferability to other settings as a result of the heterogenous natured‐broad‐spectrum DHTS which required careful results and findings interpretation	The strengths of current DHTs described by participants included improved accessibility and continuity of care, usage and treatment adherence, affordability and patient care satisfaction and trust. There were lack of coordination in systems implementation and resource allocation, lack of basic facilities and equipment, unreliable internet access and poor systems integration and interoperability
Langlois et al. [9]/Bulletin of the World Health Organization	To use data from 20 case studies to conduct a multi‐country analysis of the system‐level determinants of primary healthcare performance in low‐ and middle‐income countries	The study adopted a mixed‐methods approach and comparative research design	The PRIMASYS (primary healthcare systems) conceptual framework was adopted to guide the development and reporting of primary healthcare system case studies. Between 2016 and 2018	Even though the case studies addressed policy‐ and systems‐level determinants of primary healthcare, the nature and complexity of the evidence made it difficult to associate the performance of a primary healthcare system or an improvement in primary healthcare policy or implementation with a specific factor or initiative performance	Overall, however, the effort and investment devoted to strengthening health promotion and prevention were not commensurate with the swiftly increasing morbidity and mortality associated with noncommunicable diseases. Frequently, a primary focus on curative services undermined the comprehensiveness and continuity of primary healthcare
Ramadan et al. [25]/Access to Primary Healthcare Services in Conflict‐Affected Fragile States: A Subnational Descriptive Analysis Of Educational and Wealth Disparities	To address the literature gap by applying a conflict intensity lens to the analysis of disparities in access to essential PHC services within conflict‐affected fragile states	The study employed a descriptive cross‐sectional design	The countries were systematically selected, and the criteria include countries that had a DHS survey in the last 10 years (2010–2020), to allow for the availability of data on access to PHC using standardized indicators Conflict data were obtained using the UCDP database. UCDP is the primary global source for data on armed conflict and organized violence	The study could not account for the effect of population displacement as no trusted data were found for the geographical linkage at the household cluster level. This culminated in greatly affecting the presentation of results due to a difference in the estimated denominators	Regardless of conflict intensity, both wealth and educational disparities were observed in access to PHC services in the four studied contexts. PHC particularly receives lower funding in many low‐income conflict and fragile situations with the reliance on user fees for facility operation
Macarayan et al. [26]/Facility management associated with improved primary health care outcomes in Ghana	To examine the associations of management on service delivery process outcomes and women's experience of care using PHC facilities in Ghana	Quantitative approach	The World Management Survey (WMS) was used to quantify the management performance of PHC facilities in Ghana and the experiences of women who sought care at sampled facilities were assessed	The survey also did not capture women's expectations of care, which may have confounded their reported experiences	Elements of experiential quality that were higher in better‐managed facilities included promptness of care, issues related to provider‐client interactions (trust, communication), as well as overall user rating of quality. CHPS facilities had higher ratings on most experiential outcomes, potentially reflecting their strong focus on community engagement, but lower scores on most process outcomes
Alhassan et al. [31]/Trends and correlates of maternal, newborn and child health (MNCH) services utilization in primary healthcare facilities in the Volta region of Ghana	To explore the trends and correlates of MNCH services utilization throughout the continuum of care (CoC) in 26 primary healthcare facilities in Ho West District (HWD) of the Volta Region of Ghana	Quantitative—specifically an explorative ecological design using data from the Ghana Health Service District Health Information Management System II (DHIMSII), 2015–2017	The DHIMSII data comprise 26 primary healthcare facilities, for example, clinics, health centres and functional community‐based health planning and services (CHPS) located in urban and rural communities in the HDW. Data on MNCH service indicators were retrieved from DHIMSII	Ecological population‐based data from DHIMSII were used solely for the study with no inclusion of complementary primary data to extract personal experiences both from health providers and clients regarding service quality gaps and the implications on the use of MNCH services. The data used were 3–4 years old and may not reflect all the current realities	MNCH service utilization is significantly associated with rural–urban differentials and distribution of human and material health resources as alluded to in previous studies on Ghana and other countries. The density of midwives and other frontline health staff significantly correlated with the number of ANC visits recorded in the study health facilities over the 3 years. Poor attitudes of staff and other health system challenges remain a constraint to the utilization of MNCH services in Ghana
Ekenna et al. [37]/How ready is the system to deliver primary healthcare? Results of a primary health facility assessment in Enugu State, Nigeria	To measure policy‐to‐practice gaps and to inform the implementation of new policies to improve PHC and, by extension, UHC	A descriptive cross‐sectional study	Data collection was done through the use of a semi‐structured questionnaire	Since the study focused on primary healthcare facilities in Enugu State, Nigeria, it therefore provided insights that were directly relevant to the localized perspective, which otherwise may not be generalised to a wider population	This study found that all sampled PHC centres in Enugu State offered the different service areas at varying degrees, but none offered all the components of each service area on the day of the assessment. These gaps between the current state of PHC services in Enugu State and the recommended standards persisted despite the development of the DHS policy to reform PHC. It was also found that only 18% of surveyed PHC facilities offered mental health services, and although HIV testing services were available in 68% of PHC centres sampled, only 25% offered HIV treatment
Vallières et al. [39]/Determinants of safety climate at primary care level in Ghana, Malawi and Uganda: a cross‐sectional study	To examine the relationship between key organisational, unit and individual‐level factors and safety climate across primary healthcare centres in the selected countries	Quantitative [survey]	In‐country members of the PERFORM2 Scale project distributed a self‐administered, study‐based health worker questionnaire to 760 health workers in 138 primary health facilities in the selected countries	The cross‐sectional study could not determine causality. The varying sampling methods in different study locations made cross‐country comparisons difficult	In Ghana, individual factors explained 46% of the variance in safety climate. Job satisfaction and being clinical staff were significant predictors of higher safety climate levels. Unit‐level factors, including teamwork, job satisfaction, and supportive supervision, explained a reduced 43% of the variance In Uganda, individual factors explained 12% of the variance, with only job satisfaction predicting safety climate. The inclusion of teamwork and supportive supervision in step two increased the variance explained to 61% In Malawi, both individual and unit‐level factors significantly contributed to explaining the variance in safety climate. Job satisfaction, teamwork, and supportive supervision were strong predictors in the unit‐level factors. Organizational factors did not provide additional explanation
Haricharan et al. [49]/The role of community participation in primary health care: Practices of South African health committees	To explored South African health committees’ roles, their degree of participation and factors impacting their functioning and role	Mixed method, including a cross‐sectional survey, in‐depth interviews, focus group discussions, observations and thematic content analysis	Survey data were captured in MS Excel. Open‐ended questions were coded thematically. Interviews, focus groups, and observations were analysed using thematic content analysis	The study's data were collected between 2010 and 2012, but the authors’ experiences from subsequent work and discussions in 2014 and 2017 suggested that the findings remain consistent over time and across provinces	Health committees have limited roles in health systems, mainly focusing on raising awareness and assisting with day‐to‐day activities. Participation varies, with some members mainly receiving and recording complaints Other subthemes that were looked into include lack of clarity of role and function of health committees, limited skills, presence and attitude of facility managers, presence of ward councillors, resources and support, lack of recognition and political support and formation of health committees
Arhin et al. [35]/Effect of Primary Health Care Expenditure on Universal Health Coverage: Evidence from Sub‐Saharan Africa	To assess the effects of PHC spending on UHC and health outcomes	Quantitative, using the Grossman Health Production Model	Using panel data from 2016 to 2019, researchers analysed 34 Sub‐Saharan African countries to examine their health outcomes. The data were obtained from the World Bank, WHO, and United Nations Development Programme	Limited time series observations for PHC expenditure per capita in SSA countries hindered panel data analysis. Proxies for health outcomes lacked data on morbidity and disability, but the inclusion of three different variables ensured robust results	Results show that a 1% increase in PHC expenditure per capita leads to a 0.14 improvement in the UHC index. Higher PHC expenditure is also associated with a marginal increase in life expectancy at birth and a decrease in infant mortality rates. The study also highlights the negative impact of GDP per capita on infant mortality, as well as the contribution of access to improved water and education in reducing infant mortality rates in SSA. Overall, the study underscores the need for increased investment in PHC to enhance health outcomes and UHC in the region
Bolongaita et al. [38]/Financial hardship associated with catastrophic out‐of‐pocket spending tied to primary care services in low‐ and lower‐middle‐income countries: findings from a modeling study	To model which disease areas and conditions could pose the greatest financial risks to individuals when seeking treatment at the primary care level in low‐ and middle‐income countries (LMICs)	Quantitative	The datasets in this study were linked manually due to variations in terminologies and recorded information. NHA reports provided disease groupings based on accounting, whereas DCP3 supplied costed health services. Simulated populations were created using income data, and quintile‐specific use rates were applied based on health conditions. Only individuals using health services incurred out‐of‐pocket payments, calculated by multiplying the unit cost by disease‐specific percentages. Out‐of‐pocket payments were compared to simulated incomes using different thresholds to determine	The study was unable to account for factors at the individual level	The risk of catastrophic health expenditure (CHE), resulting from out‐of‐pocket spending on healthcare services and diseases, varied widely. On average, paediatric health issues had the lowest out‐of‐pocket amounts ($0.09), whereas cardiovascular disease (CVD) had the highest ($79.93). The poorer quintiles faced a higher risk of CHE compared to the richest quintile across all countries and diseases. Seeking non‐communicable disease (NCD) services, particularly for neurological illnesses, mental/behavioural disorders and CVD, increased the risk of CHE. Maternal health, infectious and parasitic diseases and childhood health had lower CHE risks due to lower out‐of‐pocket spending
Olabode et al. [50]/Determinants of implementation of evidence‐based practice (EBP) in clinical decision‐making among nurses in primary health care facilities	To assess the determinants of implementation of evidence‐based practice (EBP) in clinical decision‐making among nurses in primary healthcare facilities	A descriptive cross‐sectional approach	Data were collected from 266 nurses using an online survey platform (Google Forms) from October 2022 to July 2023	The small sample size prevented complete enumeration. Nurses in primary healthcare facilities in Ondo State were included, excluding those in secondary and tertiary care	In general, 73.7% of the participants demonstrated a strong understanding of evidence‐based practice in clinical decision‐making, whereas 26.3% demonstrated a deficient understanding of EBP in clinical decision‐making. Regarding the respondents’ level of EBP implementation in clinical decision‐making, 53.4% had high implementation and 46.6% had low implementation The study found that although nurses generally possessed good knowledge of evidence‐based practice, certain factors were also found to influence the application of EBP in clinical decision‐making. Over 50% of the respondents said they were not confident in their ability to assess the calibre of research and make use of online research databases

### 2.6 Analysis

We adopted a narrative synthesis consistent with the guidelines provided by Popay et al. [51], which provides a crucial framework for the narrative synthesis of quantitative evidence. Although this is a non‐quantitative method of synthesis, results could be presented in the form of narratives, tabulation or graphs, presenting patterns in reviewed data. This synthesis modality was selected because of the nature of the review question and in‐line with the assertion of Higgins et al. [52], who asserted that reviews that have no intention of evaluating the overall effect of variables or interventions across studies are best analysed and presented using non‐quantitative synthesis. For this study, the results were presented in a narrative style under key emerging themes. No meta‐analysis was done. We performed a narrative synthesis across all selected articles, looking for patterns and shared themes relating to the review focus. Because of the volume of articles selected, four of the authors worked on the data extraction and analysis, with two authors working together, separate from the others, to analyse and then compare the themes from both to formulate the six final themes that emerged.

## 3 Presenting the Result

### 3.1 Study Characteristics

After the screening process, 26 studies that met the inclusion criteria were included in this review (Figure 1). All countries in the WHO‐Afro region were included in the studies selected for this review, including Algeria (*n* = 5), Angola (*n* = 6), Benin (*n* = 7), Botswana (*n* = 6), Burkina Faso (*n* = 8), Burundi (*n* = 7), Cabo Verde (*n* = 7), Cameroon (*n* = 11), Central African Republic (*n* = 7), Chad (*n* = 7), Comoros (*n* = 7), Congo (*n* = 9), Cote d'Ivoire (*n* = 7), Democratic Republic of Congo (*n* = 8), Equatorial, Guinea (*n* = 7), Eritrea (*n* = 6), Eswatini (*n* = 7), Ethiopia (*n* = 8), Gabon (*n* = 8), Ghana (*n* = 14), Guinea (*n* = 7), Guinea Bissau (*n* = 7), Kenya (*n* = 11), Lesotho (*n* = 7), Liberia (*n* = 7), Madagascar (*n* = 6), Malawi (*n* = 7), Mali (*n* = 8), Mauritania (*n* = 7), Mauritius (*n* = 6), Mozambique (*n* = 7), Namibia (*n* = 6), Niger (*n* = 7), Nigeria (*n* = 16), Rwanda (*n* = 9), Sao Tome and Principe (*n* = 6), Senegal (*n* = 8), Seychelles (*n* = 6), Sierra Leone (*n* = 8), South Africa (*n* = 12), South Sudan (*n* = 7), The Gambia (*n* = 5), Togo (*n* = 5), Uganda (*n* = 10), United Republic of Tanzania (*n* = 7), Zambia (*n* = 6) and Zimbabwe (*n* = 7). Studies included in this review were quantitative/cross‐sectional (*n* = 16), qualitative (*n* = 8) and mixed methods (*n* = 2). Overall, the studies had low to high methodological quality (Tables 3 and 5). In most cases, each study reported on more than one country in the WHO‐Afro region, whereas a few were solely conducted in a country. All studies provided insight into the various factors influencing PHC in the achievement of UHC. These were grouped under six main themes, including ‘physical access to critical services’, ‘availability of resources’, ‘sociodemographic and cultural factors’, ‘access to data and health information’, ‘legislation and policy’ and ‘quality of care’.

**TABLE 5:** Critical appraisal of selected qualitative studies.

Authors	Q1	Q2	Q3	Q4	Q5	Q6	Q7	Q8	Q9	Q10
Kumar et al. [47]	Y	Y	Y	Y	Y	Y	U	Y	Y	Y
O'Brien et al. [48]	Y	Y	Y	Y	Y	Y	U	Y	Y	Y
Langlois et al. [9]	Y	Y	Y	Y	Y	Y	Y	U	NA	Y
Kredo et al. [44]	Y	Y	Y	Y	Y	Y	Y	Y	Y	Y
Michel et al. [45]	Y	Y	Y	Y	Y	Y	Y	Y	Y	Y
Ameh et al. [46]	Y	Y	Y	Y	Y	Y	Y	Y	Y	Y
Haricharan et al. [49]	Y	Y	Y	Y	Y	Y	Y	Y	Y	Y
Adewole et al. [32]	Y	Y	U	Y	Y	N	Y	U		
Karamagi et al. [33]	N	U	Y	Y	Y	N	Y	Y		
Bresick et al. [34]	Y	Y	Y	Y	Y	Y	Y	Y		
Arhin et al. [35]	N	U	Y	U	Y	Y	Y	Y		
Gebremichael et al. [36]	Y	Y	U	Y	Y	Y	Y	Y		
Ekenna et al. [37]	N	Y	Y	Y	Y	Y	Y	Y		
Bolongaita et al. [38]	U	U	U	Y	Y	Y	Y	Y		
Vallières et al. [39]	U	Y	Y	Y	Y	Y	Y	Y		

*Note:* This table shows the result of the critical appraisal of quantitative studies selected article using JBI (2020) guideline.

### 3.2 Physical Access to Critical Services

Physical access to essential services is a critical factor influencing PHC, particularly in the WHO‐Afro region. This encompasses functional road networks and reliable transportation systems, which influence healthcare accessibility and continuity of care [33, 34, 35, 41, 45). In addition to healthcare services, access to improved water and education has been linked to reductions in infant mortality rates in SSA [42, 44].

Several studies in this review extend this discourse by focusing on the timeliness and appropriateness of care, alongside patient wait times at health facilities [30, 32, 41, 48]. Muttono et al. [28] highlight traffic congestion as a significant barrier, hindering both patients and healthcare professionals from accessing facilities. Similarly, Adewole et al. [32] describe ‘geographical accessibility’ as the physical barriers limiting PHC access in the African region, emphasising that greater distances between residences and healthcare facilities deter individuals from seeking formal healthcare, often leading them to unsafe alternatives. These findings underscore the critical impact of physical barriers on healthcare‐seeking behaviour and the utilisation of PHC facilities.

### 3.3 Availability of Resources

The reviewed literature emphasised that the availability and allocation of resources are crucial factors influencing PHC systems in achieving UHC in the WHO‐Afro region. These resources were highlighted under three sub‐themes:

- *Human resources:* The availability and quality of human resources are pivotal for the effective delivery of PHC services. Studies highlighted inadequate staffing, burnout, and resource gaps as key challenges [47]. Kumar et al. [47] emphasised that understaffed facilities with high patient loads lead to professional burnout. Vallières et al. [39] explored 138 Primary health centres in Ghana, Malawi and Uganda and identified factors such as job satisfaction, teamwork and a safety climate as critical for improving the quality of care. In Ghana and Malawi, job satisfaction significantly influenced care quality, though its impact was less pronounced in Uganda [9]. Alhassan et al. [31] found that the density of midwives and frontline health staff correlated with the utilisation of maternal and child health (MCH) services, underscoring the importance of equitable human resource distribution.
- *Financial resources:* Financial constraints significantly affect the performance of PHC systems. Studies emphasise that increasing PHC expenditure is crucial for achieving UHC [25, 29, 35]. Arhin et al. [35] demonstrated that a 1% increase in PHC expenditure per capita leads to improvements in the UHC index and life expectancy, alongside reductions in infant mortality. They reported Seychelles as having the highest ($491.6) average PHC expenditure per capita and the Democratic Republic of Congo as having the lowest ($10.2) in 2018. The UHC index ranged from 30.53 (Nigeria) to 79.49 (South Africa), and life expectancy at birth ranged from 51.59 years (Central Africa Republic) to 74.52 years (Mauritius). Seychelles had the lowest infant mortality rate (12.3), whereas Sierra Leone had the highest (89.7). Low‐income mothers often rely on herbs or alternative care due to financial barriers [38, 45]. Langlois et al. [9] and Simen‐Kapeu et al. [29] stress that low budgeting remains a key limiting factor for PHC in LMICs, particularly in most African countries. Out‐of‐pocket healthcare expenses, as highlighted by Bolongaita et al. [38], Bresick et al. [34] and Ameh et al. [46], further exacerbate financial inequities, with costs varying widely based on the conditions treated. The need for increased financial investment and reduced dependency on out‐of‐pocket payments is critical to enhancing health outcomes.
- *Infrastructural resources:* Inadequate infrastructure poses significant challenges to PHC delivery. Ekenna et al. [37] and O'Brien et al. [48] underscore the importance of infrastructure development, including functional health facilities equipped with reliable water, sanitation and diagnostic tools. WHO [3] emphasises the role of physical architecture in influencing both staff efficiency and patient satisfaction. Assan et al. [41] highlighted the limited use of digital technologies in community‐based health planning. Difficulties in procuring medical supplies and delayed test results further constrain PHC services [30, 32, 33, 42, 43]. Investments in infrastructure, particularly in underserved regions, are essential to bridge these gaps and support comprehensive healthcare delivery.

### 3.4 Sociodemographic and Cultural Factors

This review highlights cultural and sociodemographic factors as critical influences on decisions to seek medical care and utilise primary healthcare facilities. Religious and cultural beliefs significantly shape health‐seeking behaviours, necessitating targeted social mobilisation to maximise the potential of Primary health centres in the WHO‐Afro region [3, 33, 34, 41]. Ramadan et al. [25] emphasised wealth and educational disparities as key factors, whereas Kredo et al. [44] highlighted the impact of limited social capital and community capacities on the Primary health centres.

Simen‐Kapeu et al. [29] identified inadequate community engagement and poor service integration across 14 African countries, hindering PHC effectiveness. Similarly, Haricharan et al. [49] explored the roles of South African health committees, noting their focus on raising awareness and supporting facility operations. However, community participation was limited by unclear committee roles, lack of skills, minimal support and insufficient political backing. Tumusiime et al. [43] advocated for multisectoral collaboration to revitalise and sustain Primary health centres, critical for achieving UHC.

### 3.5 Access to Data and Health Information

Access to health information and data emerged as an important factor influencing the effectiveness of PHC services. Barriers such as staff shortages, inadequate remuneration and transportation constraints hinder the availability of healthcare professionals [28]. Information platforms like digital health technologies (DHTs) have been shown to improve care quality, support health education and promote UHC [37, 48]. However, challenges, including poor digital literacy and health inequalities, often limit the utilisation of DHTs [48].

Simen‐Kapeu et al. [29] reported that approximately 50% of African countries face a lack of community‐level data, poor data quality and limited capacity to analyse and apply this data for decision‐making. These gaps underscore the need for robust information management systems. Tumusiime et al. [43] advocated for transitioning PHC systems from fragmented models to integrated frameworks, ensuring effective implementation and information exchange to achieve UHC. Ray and Mash [42] highlighted the essential role of information technology in enhancing PHC effectiveness. Further, Olabode et al. [50] identified a knowledge deficit and limited access to data and health information, which hinders the implementation of evidence‐based practices (EBPs) within Nigerian Primary health centres, whereas Kumar et al. [47] reported staff training gaps, particularly in mental health and psychosocial support in Nairobi, Kenya.

### 3.6 Legislation and Policy

Effective policy design, execution and international support have been identified as critical factors influencing PHC systems by various authors in this review [27, 33, 34, 41, 44, 45, 48]. Key policy and legislative stakeholders, including national authorities, political leaders, community figures and public–private partnerships, play vital roles in implementing community‐oriented PHC approaches. Langlois et al. [9] discovered that community‐based services are the cornerstone for achieving UHC, an area significantly underdeveloped in LMICs.

Assan et al. [41] highlighted a lack of understanding of community‐based health planning and services among stakeholders, including government institutions and community leaders, as a significant barrier. Contextual challenges such as shifting political priorities and stalled policy adjustments further threaten the sustainability of these initiatives. Kredo et al. [44] identified the absence of agreed systems for clinical practice guideline development, a national community engagement strategy and mechanisms to ensure accountability among local stakeholders.

Kumar et al. [47] revealed gaps in policy awareness among healthcare workers in Kenya, especially regarding mental health, noting the lack of standard protocols for facility‐based or public mental healthcare information. Olabode et al. [50] reported limited research capacity among Nigerian nurses due to the lack of policy and guidelines to create an enabling environment, which undermines the integration of evidence‐based practices in clinical decision‐making at Primary health centres. Ray and Mash [42] reported a disconnect between public health policies and the sociocultural, religious and political contexts of communities, which stands as a significant barrier to successful intervention adoption.

### 3.7 Quality of Care

Quality of care has been identified among several factors influencing the efficiency and effectiveness of PHC systems. This critical factor was further diffused by several authors in this review to include promptness of care, waiting time, communication, user ratings of quality, trust and perceived quality of care [26, 28, 48]. Bresick et al. [34] and Ameh et al. [46] highlighted additional challenges such as limited service availability after working hours and on weekends, poor continuity of care in chronic disease management, drug stockouts, unprofessional staff conduct and long waiting times, which compromise service quality.

Systemic issues, including disease shock, the COVID‐19 pandemic, poor adaptability to past experiences, inadequate health management information systems and limited healthcare expenditure, were noted in some of the selected articles ([9, 28, 37, 43]. Similar concerns, including gaps in the implementation of policies, staff skill deficits and unsatisfactory user experiences due to poor staff attitudes, were noted by Karamagi et al. [33] and Ekenna et al. [37]. Langlois et al. [9] reported that the primary focus on curative services over health promotion and prevention in most LMICs undermines the comprehensiveness and continuity of PHC services, further affecting user satisfaction and trust. A study in Enugu State, Nigeria, by Ekenna et al. [37] revealed significant service gaps, with only 18% of Primary health centres offering mental health facilities, 68% providing HIV testing and just 25% offering HIV treatment. None of the surveyed facilities provided all recommended components of care, highlighting disparities between policy frameworks and actual practice. Healthcare worker resilience influences their capacity to maintain service quality amidst other challenges [33, 42, 43].

## 4 Discussion

This study identifies, evaluates and synthesises evidence on the factors influencing PHC in achieving UHC in the WHO‐Afro region.

The study shows that physical barriers such as inadequate road networks, transportation challenges and traffic congestion critically affect access to essential healthcare services. Additionally, these geographical factors, including greater distances to the nearest Primary health centre, influence the healthcare‐seeking behaviour of people within the community, deterring formal care and reliance on unsafe alternatives. This is consistent with other authors’ findings. For instance, Syed et al. [53] highlighted transportation challenges as a key healthcare access barrier leading to delayed care, missed appointments and poor management. Similarly, Cochran et al. [54] reported similar findings, which corroborate the findings from this review. Physical barriers have been emphasised over the years as a key factor influencing PHC in achieving UHC. The findings of this study demonstrate that this challenge continues to persist in the African region. Hence, there is a need to consider this when planning future interventions.

This review also reveals critical challenges relating to financial, infrastructural and human resources within the PHC system in the WHO‐Afro region. Inadequate funding deters the availability of equipment, medicines and health commodities, especially in government facilities. This is made worse by out‐of‐pocket healthcare spending, leading to a rise in catastrophic health expenditure risks. Inadequate infrastructure or its poor state, including health monitoring gadgets, power supply, unreliable water and sanitation, hinders quality care and patient satisfaction. Staff shortages due to burnout, low motivation, poor remuneration and limited equipment to work with have all negatively contributed to the ineffective state of the Primary health centres in the WHO‐Afro region. These concerns relating to infrastructure, financial and human resources have recently been highlighted in major scholarly discussions [50, 55, 56, 57]. The three factors, financial, infrastructural and human resources, are intricately interwoven with inadequate financing, serving as a pervasive issue influencing all other resource dimensions. Sufficient funding is required to equip PHC facilities within the community to adequately meet the population's healthcare demands. Additionally, financial constraints worsen staffing challenges, as healthcare workers require both a conducive work environment and adequate remuneration to remain motivated. Several studies have reported that most healthcare workers in the WHO‐Afro region often refuse to work at Primary health centres, citing the rural and often dilapidated state of these facilities, coupled with concerns that such roles may be perceived as inferior to positions in state or teaching hospitals [58, 59, 60, 61, 62].

This study's findings reveal that cultural and sociodemographic factors, including religious and educational disparities, significantly influence decisions to seek medical care and use PHC facilities. Effective community participation and social mobilisation are critical for maximising the potential of PHCs, and intersectoral collaboration is suggested as a vital strategy for achieving UHC in the WHO‐Afro region. Cultural and sociodemographic factors have been noted in the literature as key factors influencing the effectiveness of PHCs, which resonate with the findings of this review [59, 63, 64, 65]; WHO [3]. LMICs, particularly African countries, are characterised by very rich cultural diversity [66], which significantly shapes health perceptions and decision‐making processes. For instance, some cultural or religious beliefs discourage individuals from seeking medical care at PHC facilities, undermining healthcare access [67, 68]. This review highlights that these sociocultural and demographic factors critically influence healthcare‐seeking behaviour and pose significant challenges to achieving UHC.

The findings of this study identify access to health information and data as key factors influencing the PHC system. However, workforce shortages, inadequate remuneration and transportation barriers constrain these factors, creating an interconnected web of challenges. This interdependence underscores the importance of a holistic approach to addressing gaps, where resolving one issue could unlock solutions for others. Supporting this perspective, studies in Iran and China similarly emphasised the role of comprehensive strategies in addressing PHC challenges [5, 69]. Digital health technology (DHTs) has demonstrated potential for improving access to health information and care quality [48, 57, 70]. However, this review shows that although DHTs enhance care quality and health promotion, their utilisation is hindered by poor digital literacy and health inequities.

This study's findings also highlight policy design, effective implementation and international support as critical factors influencing PHC system. However, challenges, including stakeholder disengagement, lack of accountability framework, poor community engagement and limited understanding of community‐based initiatives, deter the sustainability and efficiency of PHC services. Simen‐Kapeu et al. [29] corroborated these findings, emphasising the critical need for locally established mechanisms to enhance stakeholder accountability. Similarly, these findings align with authors who underscored policy and political leadership as very crucial factors that must be considered to revitalise the PHCs [5, 11].

The findings from this review underscore other factors influencing PHC effectiveness, including timeliness of care, user trust and perceived quality. Barriers such as long waiting times, unprofessional staff conduct, drug shortages and policy‐to‐practice gaps further complicate the delivery of comprehensive PHC services. Additionally, gaps in the comprehensiveness of service, such as limited mental health facilities and inconsistent delivery of essential services, despite policy reforms. According to Hanson et al. [11], PHC is crucial to addressing the community's basic healthcare needs, including the prevention and treatment of non‐communicable diseases and ensuring accessibility even in the smallest community units. This positions PHC as an essential component of an effective healthcare system, critical for achieving UHC. However, evidence has shown the formidable challenges of achieving UHC in the African region, where socioeconomic and systemic barriers hinder progress [56, 59, 71]. The findings of this study suggest that addressing these multifaceted barriers requires targeted systemic interventions to enhance the efficiency and equity of PHC system. This aligns with the WHO's [72]  strategic direction for nurses and midwives in the area of service delivery, asserting that the revitalisation of PHCs is pivotal for achieving UHC and SDG3. By strengthening service delivery at the PHC level, these objectives can be advanced, ensuring equitable access to essential health services.

## 5 Study Limitation

The systematic search for pertinent literature focused on the factors influencing PHC systems in the WHO‐Afro region. This scope may have excluded high‐quality studies from non‐African contexts. The critical appraisal for methodological quality also limited the number of eligible studies for inclusion. In addition, the choice of keywords, the searched database, the data extraction process and the analysis may have influenced the search outcome. At least four authors independently conducted searches and cross‐validated the results to mitigate these limitations. Furthermore, two authors participated in key stages of the review process, with the entire manuscript reviewed and approved by all authors to ensure rigour and consistency.

## 6 Conclusions and Implications

This review summarises evidence on the factors influencing the PHC system in achieving UHC in the WHO‐Afro region. Primary health centres, which are essential pillars of the healthcare system, have experienced numerous setbacks since their implementation, particularly in Africa. Key factors identified were summarised under themes, including physical access, quality of care, availability of resources, access to data and health information, sociocultural and demographic factors and legislation and policy. These interconnected factors underscore the need for a holistic approach to optimise PHC effectiveness and drive progress towards achieving UHC. To enhance PHC systems in underserved regions, particularly the WHO‐Afro region, a multifaceted approach is essential. Prioritising the integration of human, financial and infrastructural resources is critical to reducing inequities, advancing UHC and improving health outcomes. Addressing maternal education, income disparities and cultural and sociodemographic barriers requires community‐driven strategies, intersectoral collaboration and targeted advocacy. Strengthening PHC also demands investment in training, data utilisation and equitable access to digital technologies. Finally, sustainable policy frameworks and accountability structures must be established to ensure effective, equitable and resilient PHC services globally.

## Author Contributions

**Emmanuel O. Adesuyi**: conceptualisation, methodology, database search and screening, analysis and synthesis, writing the original draft and editing, overall supervision. **Opeoluwa O. Olabode**: conceptualisation, methodology, database search and screening, data extraction and critical appraisal, writing the original draft and editing. **Oluwatosin C. Olarinde**: conceptualisation, methodology, writing the original draft and editing. **Blessed O. Oyama**: conceptualisation, methodology, writing the original draft and editing. **Inioluwa O. Aderemi**: conceptualisation, methodology, writing the original draft and editing. **Gloria O. Alao**: database search and screening, data extraction and critical appraisal, analysis and synthesis, writing the original draft and editing. **Bose C. Ogunlowo**: database search and screening, data extraction and critical appraisal, analysis and synthesis, writing the original draft and editing.

## Ethics Statement

The authors have nothing to report.

## Conflicts of Interest

The authors declare no conflicts of interest.

## Data Availability

The authors have nothing to report.
